# “Broadcast your gender.” A comparison of four text-based classification methods of German YouTube channels

**DOI:** 10.3389/fdata.2022.908636

**Published:** 2022-09-14

**Authors:** Lena Seewann, Roland Verwiebe, Claudia Buder, Nina-Sophie Fritsch

**Affiliations:** Faculty of Economics and Social Sciences, University of Potsdam, Potsdam, Germany

**Keywords:** text based classification methods, gender, YouTube, machine learning, authorship attribution

## Abstract

Social media platforms provide a large array of behavioral data relevant to social scientific research. However, key information such as sociodemographic characteristics of agents are often missing. This paper aims to compare four methods of classifying social attributes from text. Specifically, we are interested in estimating the gender of German social media creators. By using the example of a random sample of 200 YouTube channels, we compare several classification methods, namely (1) a survey among university staff, (2) a name dictionary method with the World Gender Name Dictionary as a reference list, (3) an algorithmic approach using the website gender-api.com, and (4) a Multinomial Naïve Bayes (MNB) machine learning technique. These different methods identify gender attributes based on YouTube channel names and descriptions in German but are adaptable to other languages. Our contribution will evaluate the share of identifiable channels, accuracy and meaningfulness of classification, as well as limits and benefits of each approach. We aim to address methodological challenges connected to classifying gender attributes for YouTube channels as well as related to reinforcing stereotypes and ethical implications.

## Introduction

Every day, thousands of people around the world share their homes, thoughts, and activities on social media platforms such as YouTube. This online self-representation provides an extensive and accessible resource for research in various disciplines, focusing on different aspects of YouTube as a platform. Among other things, YouTube is discussed as a cultural phenomenon (Boxman-Shabtai, 2018; Burgess and Green, 2018). Especially among younger age groups, the more than 30 million YouTube channels worldwide have become a primary source of social, cultural, and political information, whose relevance is significantly higher than that of traditional media formats such as newspapers and TV (Mitchell et al., 2018; Litvinenko, 2021). Other authors study the functions of the platform algorithm and examine the impact it has on consumers and producers (Rieder et al., 2018; Bishop, 2020; Bryant, 2020). Most of the existing studies deal with various aspects of the presence and activity of YouTubers within the platform. The range of topics is wide and includes economic (mis)success (Postigo, 2016; Soha and McDowell, 2016; Duffy, 2020), political activism among YouTubers (Ekman, 2014; Sobande, 2017), the popularity and content of YouTube channels (García-Rapp, 2017; Ladhari et al., 2020), or the use of emotional labor, i.e., creating closeness and authenticity through which the attention and attachment of viewers is to be obtained (Berryman and Kavka, 2018; Raun, 2018; Rosenbusch et al., 2019). This research employs a wide range of methods. A large number of studies use qualitative interviews (Choi and Behm-Morawitz, 2017; Sobande, 2017; Bishop, 2019), video ethnographic methods or netnographic methods (García-Rapp, 2017; Mardon et al., 2018), qualitative content analysis, or discourse analysis (Montes-Vozmediano et al., 2018; Scolari and Fraticelli, 2018; Lewis et al., 2021). Quantitative analysis, webscraping, or Machine Learning-based methods are less frequently used in current YouTube research (Zeni et al., 2013; Schwemmer and Ziewiecki, 2018; Kalra et al., 2019; Obadimu et al., 2019), although the already quantified digital setting of the platform seems to lend itself to such an approach (Munger and Phillips, 2022).

This observation marks the starting point for this paper, in which we aim to use a random sample of German YouTube channels and apply four different classification methods in order to assess the gender of channel creators. We concentrate on the classification of gender for the following reasons: (1) gender shapes how people make sense of themselves, their social relationships, their networks, and their professional activity. A person's gender, once it becomes visible online, is important to describe people's behavior and is relevant to explaining mechanisms of inequality on social media platforms and beyond–this might even mimic or exaggerate gender inequalities that already exist in the offline world (Molyneaux et al., 2008; Wagner et al., 2015; Muñoz Morcillo et al., 2019). (2) We need to examine the contexts in which the absence of women in digital narratives, and the often stereotyped expression of them, form a constitutive part and reproduce a patriarchal system of imaginaries associated with prestige, reason, and power (Regueira et al., 2020). We also have to highlight which contexts provide new potential to change existing social hierarchies, tackle traditional boundaries to promoting mariginalized groups, and therefore could even play a role in turning women's or individuals with non-binary gender identities' talk into voice (Sreberny, 2005; Molyneaux et al., 2008). (3) However, when we focus on previous research, it becomes apparent that despite the existence of an enormous potential introduced by this data source, the social structure of the YouTube community is still ambiguous and not properly explored. The lack of personal information (such as gender, age, education, or ethnicity) is intriguing, because we know that individual characteristics have a significant impact on who creates online content in the first place, but equally important is which content is produced and why it is (not) widely circulated (Haraway, 2006; Regueira et al., 2020; van Dijk, 2020).

From a practical point of view, the YouTube API easily provides access to comprehensive data (such as content of videos, number of views, inter-personal comments etc.). Using text strings from channel names and descriptions we aim to distinguish male, female, and multi-agent presentations^1^ We will evaluate and compare four classification methods in terms of the performance and meaningfulness of classification, as well as the resource efficiency of each approach.

The remaining sections of the paper are structured as follows: chapter two offers a summary of previous research on YouTube by focusing on frequently used methods in this realm. Thereafter, in chapter three we describe our dataset and provide precise information about the classification methods we use. We compare our reference data set gained through a multi-platform research to (i) a classification survey, (ii) a dictionary-based method, (iii) an algorithmic classification approach using gender-api.com, and (iv) a machine learning approach that uses Multinomial Naïve Bayes (MNB). In chapter four we present the performance of each method, focusing on accuracy, precision, and recall, as well as the Brier score and combined weighted classifier (Performance) (Kittler et al., 1998; Yan and Yan, 2006; Filho et al., 2016; Kalra et al., 2019; Weissman et al., 2019). Moreover, we discuss limits and benefits, and give some examples for misclassifications that showcase the meaningfulness of the acquired results (Meaningfulness) (similar as in studies such as Wu et al., 2015; Hartmann et al., 2019; Grimmer et al., 2021). The chapter concludes with remarks on benefits and challenges of using YouTube data. In chapter five we discuss our results and offer some concluding remarks.

## State of the art

Advances in computational methods have opened up new possibilities in using social media data for social science research in the last decades. As a result, a multitude of text-based classification methods have been established in recent years. In the following, we want to give a short insight into the current use of a variety of text-based classification methods within the social sciences, and illustrate their application to different topics and methodological challenges as they present themselves today.

A large number of studies, especially in the beginning of web-related research, have employed classification surveys to assess information that is difficult to access for automated methods, such as viewing experiences and emotions (Hoßfeld et al., 2011; Biel and Gatica-Perez, 2013). MoorMoor et al. (2010, p. 1539), for example, used several questionnaires to assess flaming on YouTube, defined as displaying hostility by insulting, swearing, or using other offensive language. Konijn et al. (2013) conducted a survey in a mixed-method study (that also featured experimental designs) of social media preferences and moral judgments among a younger YouTube audience. With respect to employed methods, the study of Fosch-Villaronga et al. (2021) is relevant as well, with the results of a survey among Twitter users showing that platform algorithms can (re-)produce inaccurate gender inference. The initial popularity of classification surveys has seen a decrease since the establishment of automatic classification approaches, which do not rely on the costly involvement of respondents. However, to this day, especially when complex classes such as gender identities are concerned, the use of surveys is an established method in classifying social media data.

Dictionary-based text classifications mark a shift toward automatic classification approaches. Their use is often resource intensive, because it requires the establishing of copious dictionaries that researchers share across generic domains or obtain from administrative or survey data (Hartmann et al., 2019, p. 23). The method is most widely used in research where multiple generic lexicons exist, such as in sentiment analysis (Feldman, 2013; Devika et al., 2016; Zad et al., 2021) which has produced an extensive literature. In other fields, dictionary methods have gained less prominence, since they underperform in comparison to algorithmic approaches and machine learning models (e.g., González-Bailon and Patoglou, 2015; Hartmann et al., 2019). Algorithmic classification approaches that use APIs of websites like, gender-api.com, genderize.io, NamSor, or Wiki-Gendersort are also quite common in recent studies (Karimi et al., 2016). These services offer automatic classification by comparing various types of character strings to large privately owned databases. In recent research this cost-effective method is used, for example, to explore the gender gap in scientific publications, or the identification of gender diversity in groups of knowledge production (Larivière et al., 2013; West et al., 2013; Fox et al., 2016; Giannakopoulos et al., 2018; Sebo, 2021). Although their emphasis lies in the analysis of gender-inference based on names, similar third-party APIs also exist for the detection of nationalities (e.g., https://nationalize.io/) or age (e.g., https://agify.io/).

Finally, more complex supervised machine learning approaches tackle a broader variety of goals when applied to YouTube data, such as classifying the content of YouTube videos (Kalra et al., 2019), or estimating the political ideology of channels (Dinkov et al., 2019). Most of them use video content (Kalra et al., 2019; Ribeiro et al., 2020) or viewer comments (Hartmann et al., 2019) as their main data source. When text from YouTube and other social media platforms is concerned, most approaches use Random Forest Classifiers (Kalra et al., 2019), Naïve Bayes (Hartmann et al., 2019), Support Vector Machines (Pratama and Sarno, 2015), K-nearest neighbor (Agarwal and Sureka, 2015) or a combination of multiple approaches (Park and Woo, 2019). For example, in a recent study Hartmann et al. (2019) compared 10 text classification approaches across 41 social media datasets and found that Naïve Bayes is well-suited for YouTube data and known to be fast, easy to implement, and computationally inexpensive (Filho et al., 2016; Kowsari et al., 2019). Various studies point out that classifying sociodemographic information in this way also holds questions of research ethics, such as the dangers of gender stereotyping through incorrect inferences of social media data, as was pointed out by Fosch-Villaronga et al. (2021). Some of the adverse consequences they highlight are statistical or legal discrimination, stigmatization, reinforcing of gender binarism, or self-identity issues.

It becomes clear that the range of available methods to tackle social media text classification is wide and ever-growing as more researchers take these information sources into account. However, it also becomes increasingly hard to gain an understanding of the benefits and limitations that these methods can offer. A number of methodological studies dedicated to the systematic comparison of methods already exists (González-Bailon and Patoglou, 2015; Jindal et al., 2015; Hartmann et al., 2019; Kowsari et al., 2019). However, this methodological literature is pioneered in disciplines such as computer science, often concerned with specific technical challenges. Adaption of these methods to social scientific research, and a systematic understanding of the quality and bias within these classifications, in terms of social issues, is still lacking, and marks the motivation for the study at hand.

## Materials and methods

### Dataset

The data we utilize in this paper consists of 200 German YouTube channels that we collected in March of 2020 using the YouTube API. Channels were selected with the help of a free of charge website (www.channelcrawler.com) that has since been made subject to a fee. In 2020, the website allowed identification of YouTube channels with more than 1,000 views in total. In order to randomize our sample, 200 channels which had uploaded a video most recently on March 17^th^ of that year were chosen. This procedure avoided picking channels based on their topic, prominence, or number of views, which is common in some (qualitative) studies (Jerslev, 2016; Fägersten, 2017; García-Rapp, 2017; Duguay, 2019; Wegener et al., 2020). The API provided us with information such as the YouTube channel name, the channel description, the number of views, as well as information on the 61,071 videos uploaded by the channels. We restricted our sample to 200 cases, because we used two resource-intensive and time-consuming research strategies (a multi-platform research and one online survey), for which we had a limited number of staff at the University of X.

Information we could not gather using the YouTube API was filled in using a multi-platform research strategy (Jordan, 2018; Van Bruwaene et al., 2020). At this stage, we looked up sociodemographic characteristics (such as gender, age, education, and ethnicity) on the web, including Facebook and Instagram profiles, Twitter accounts, Google and Wikipedia records, or other YouTube channels. This multi-platform research strategy was done by one female and one male researcher in May 2020. Each person classified 100 cases of the reference data set. In order to check for interrater reliability, we selected 40 cases which were processed by these researchers; results revealed no inconsistencies. Both proceeded in three steps. First, we used the *N* = 200 YouTube channels to extract available sociodemographic information from the channel descriptions, profile pictures, or other video content. This first step allowed us to classify gender in about two-thirds of all cases, age and ethnicity for roughly one-third, and education for roughly one-quarter of all channels hosts. In a second step, we looked at the Facebook, Instagram, and Twitter pages linked on these YouTube channels to find information that was missing. In a final step, we used Google and Wikipedia data to identify additional sociodemographic characteristics, if necessary. This course of action allowed us to fill in all information available, and therefore serves as our reference data set. One important distinction was whether the channel featured an individual YouTuber, or a form of multi-agent-channel (pair, group, organization). For those channels that were representative of an individual, the variables assigned included the gender of the YouTuber (female, male, non-binary) as well as age, ethnicity and educational level (if available).

The final reference data set consists of 26 (13%) female and 129 (65%) male individuals, as well as 39 (20%) multi-agent channels^2^. One channel (<1%) within our dataset featured a person with a self-declared non-binary gender identity (specifically identifying as a demiboy, a person with mostly male characteristics). Five channels (2%) could not be assigned due to missing information on gender categorizations and therefore classified as NA (not available). Aside from gender, the sample presents itself as diverse also in terms of age, ethnicity and video content. In the final reference data set, the average age of the YouTubers is 29 years, ranging from 11 to 63 years. In terms of ethnicity, a migration background was estimated in 15% of the cases (based on country of birth and surnames). The dataset consists of a large range of channels, including political channels, car enthusiasts, religious channels, gaming, beauty and lifestyle channels, local news, travel, and channels linked to TV shows. On average, each channel had uploaded 306 videos and collected 5 million views overall. However, the inequality within this distribution is significant, amounting to a Gini-coefficient of 0.94 with regards to views^3^.

### Classification methods

In this paper, we use four different classification methods to infer the gender of YouTubers from text information, and to compare the quality and limits of these approaches for social science research.

*First*, we conducted a classification survey, in which respondents were asked to identify gender identities based on text presented to them. An online questionnaire was generated using SoSci Survey (Leiner, 2019) and distributed among ten members of staff at the University of ^*^X. The respondents varied by age, gender, education, and familial status, and were told to each classify about 20 randomly selected YouTube channels. In a first step, respondents were shown the name of the channel and its description. They were asked to categorize the channels by type of YouTuber into one of four categories: individual, group, organization, or other. When a channel was classified as “individual,” respondents were asked to classify the gender by the following question: “Based on the name and the description of the channel, can a statement be made about the gender of the person?”. Answer options included the following categories: Women, men, non-binary, no statement possible. The questionnaire also assessed the gender composition of multi-agent channels, and estimated the age and education background of YouTubers, categories which are not the central to the present paper.

*Second*, we used a dictionary based approach (Jaidka et al., 2020) to classify the channels. In our case, gender classification was made accessible by inferring the given names of YouTubers from their channel names and channel descriptions. As a reference list, we used the second edition of the World Gender Name Dictionary (Raffo, 2021), which includes 26 million records of given names, including 62,000 names for Germany. The dictionary classification method compares all words of the channel names and channel descriptions against this database and counts the number of female and male names identified in the text. To classify multi-agent channels as a third category we defined a list of German key words for “we,” “team,” “institute,” “organization,” “firm,” “company,” “group,” “us,” “our.” Thus, we were able to classify and count the number of female names, male names, and multi-agent identifiers. When no identifiers whatsoever were found, the channels remained unclassified. When both female and male names were present, but no multi-agent identifier, the majority category guided classification. In cases where the same amount of female and male names were found, but no multi-agent identifier, the channel remained unclassified.

*Third*, we applied an algorithmic classification approach using gender-api.com. The R implementation of this algorithmic classification enabled us to predict the gender of a YouTube channel creator. The method estimates the gender based on a character strings and given names (Wais, 2016), referring to a database. This database of gender-api.com is built on continuous scanning of public records, registry data, and public profiles and their gender data on major social networks, and offers 6,084,389 records in total. The website is free of charge if queries do not exceed a certain level per month. It displays the number of data records examined in order to calculate the response and releases probabilities, indicating the certainty of the assigned gender.

*Fourth*, we deployed a machine learning approach using Naïve Bayes Classifiers for small samples (see Filho et al., 2016; Hartmann et al., 2019)^4^. The text preprocessing for this step consisted of transforming all words to lower cases, removing URLs and separators (such as hyphens), as well as punctuation and single digits. Common stopwords (such as “or,” “and,” “he,” “she,” “we”) were retained, since previous studies find that the removal of stop words lowers gender classification accuracy (Yan and Yan, 2006). These words also proved key to identifying multi-agent YouTube channels, similar to our dictionary approach^5^. Another important source of information was the gender specific use of Emojis, which is in line with recent studies (Wolny, 2016). Although the diverse Unicode representations of Emojis took some effort to account for in text preprocessing, these symbols proved very important in our classification. Finally, we did not conduct stemming of words in accordance with previous critiques that suggest the loss of important information (e.g., Dave et al., 2003; Bermingham and Smeaton, 2010). To estimate the out-of-sample accuracy, we split the dataset into a training set and a hold-out test set (80 vs. 20% of the data)^6^. The MNB model was trained on the training set, and the performance estimated on the test set. Laplace smoothing (a = 1) was applied as a regularization method, to avoid the zero-observation problem. Furthermore, since the categories in our dataset are not equally distributed, the prior probability of categories was factored into the model. However, there is still a large margin of error in randomly splitting a test and training sample. To better estimate the generalized performance of our method on YouTube data we applied the aforementioned procedure to five different splits of test/training data within our sample, and estimated the average performance across all five splits, also called outer fold cross validation (Parvandeh et al., 2020). This procedure also helped us to compare the output of machine learning algorithms to the other classification methods and identify particularly challenging cases.

### Information used in classification

As Table 1 illustrates, the classification methods described above rely on different sources of information. To begin with, the classification survey presented the channel name and description to the respondents, and asked them whether they could estimate the author's gender on the basis of this information. The dictionary method also considers the channel name and description, whereas with gender-api.com we based their classification only on the channel name. API approaches can be extended using the channel description as well, but these longer texts also introduce a lot of noise that can be misinterpreted as names. Finally, the MNB model uses the channel descriptions as bag of words, and finds commonalities in words used across genders. In this case, the addition of the channel name to these methods would be possible, but additional information is likely minimal unless the channel names follow a certain pattern, or are given more weight in comparison to the description^7^.

**Table 1:** Information regarded in single classification methods.

	**Reference data set**	**Classification survey**	**Dictionary method**	**Gender Api**	**MNB**
Channel name	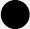	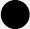	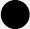	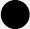	.
Channel description	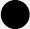	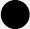	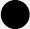	.	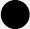
Channel profile picture	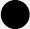	.	.	.	.
Video content	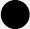	.	.	.	.
Information from other platform (e.g., Twitter)	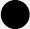	.	.	.	.

Source: own illustration.

### Evaluation

We evaluated the performance of each text classification method in terms of four parameters: (1) First, we discussed each classification method in terms of the degree to which these approaches come to the same classification result as our reference data. Four measures were evaluated and explained using the following examples (Yan and Yan, 2006; similar as in Filho et al., 2016; Kalra et al., 2019; Weissman et al., 2019): *Accuracy*, which displays the ratio of correctly predicted women within all observations. Accuracy is well-suited to evaluate the overall performance of the methods, but also has some limitations (Kowsari et al., 2019). In datasets where the categories are unbalanced (one including more cases than the others), it is wise to include precision and recall as well. *Precision* (also known as positive predictive rate) measures how many of the channels we predicted as female, were actually female. Precision is most important when false positives are to be avoided, for example, when men should not wrongly be classified as women. To see how precise the overall method was, we used macro-averaged precision (Murphy, 2012, p. 183), which average the precision over all classes. *Recall* [also known as sensitivity or true positive rate (Murphy, 2012, p. 181)] quantifies how many, out of all actual women, were labeled as female. Recall is especially important when false negatives are to be avoided, for example, when we want to minimize the women overlooked by our classification. Again, Macro-recall averages the performance between all classes to evaluate the models as a whole. Finally, the *Brier score* is reported (Brier, 1950) for those methods that compute probabilities for a channel belonging to each of the classes. The Brier score takes into account how close the predictive probability was to the correct outcome, in our case if we assigned a female led channel the probability of being 60 or 95% female. The more accurate the prediction is, the closer the Brier score is to zero. The Brier score also has the advantage of handling predictions of multi-class classifications, making it useful in the application of gender prediction (including multi-agent channels). (2) Second, we also assessed the limits and benefits of the four methods to the study of YouTube data. This should help researchers to evaluate whether the method is realizable for them, and which trade-offs exist between those methods. (3) Third, the meaningfulness of the achieved gender classifications is an additional aspect we considered. This includes discussions of misclassifications, hard to reach groups and similar issues (see Fosch-Villaronga et al., 2021). (4) Fourth, we tackled ethical challenges that arise in our study, such as the reproduction of stereotypes and consent to participate in research.

## Results

### Performance

The classification survey approach shows the second-best overall performance in Table 2, with an accuracy, precision and recall around 60%. Since hand-coded survey classifications are useful as training data for machine learning approaches, they can play a big role in determining the quality of follow up methods used (e.g., Brew et al., 2010). In our case, no probability-based Brier score was available for this method, since each channel was classified only once. However, allowing for multiple classifications, and assessing the interrater-reliability and Brier score based on the probability of classifications could increase the performance of this method.

**Table 2:** Accuracy, precision and recall of classification methods.

		**Classification**	**Dictionary**	**Gender**	**MNB**	**Average MNB**	**Weighted**
		**survey**	**method**	**API**	**(*n* = 40)**	**(5 folds, *n* = 200)**	**vote**
Male	Accuracy	0.688	0.598	0.593	0.675	0.718	**0.779**
	Precision	0.972	0.838	0.747	0.869	**0.872**	0.801
	Recall	0.535	0.477	0.569	0.667	0.746	**0.876**
Female	Accuracy	0.925	0.754	0.879	0.800	0.869	**0.894**
	Precision	0.824	0.274	0.533	0.222	0.228	**0.619**
	Recall	0.538	0.538	**0.615**	0.667	0.500	0.500
Multi-Agent	Accuracy	0.905	0.764	-	0.900	**0.849**	0.834
	Precision	0.702	0.390	-	0.571	0.520	**0.609**
	Recall	0.868	0.421	-	0.800	**0.620**	0.368
NA	Accuracy	0.678	0.819	0.573	0.975	**0.950**	0.709
	Precision	0.047	0.030	0.200	1.000	0.250	**0.525**
	Recall	0.500	0.200	0.326	0.500	0.250	**0.478**
Total sample	Accuracy	0.598	0.467	0.522	0.675	0.698	**0.709**
	Macro-Precision	0.636	0.383	0.494	0.658	**0.578**	0.525
	Macro-Recall	0.610	0.409	0.503	0.666	**0.485**	0.478
	Brier score	-	-	0.158	0.040	0.061	-

Source: own calculations; N = 200. Accuracy is the ratio of correctly predicted cases within all observations. Precision is the ratio of all correctly predicted cases within all prediction in one class. Recall is the ratio of correctly predicted cases within all cases that actually belong to said class. The Brier-score shows the accuracy of the probabilistic prediction. Bold values represent the highest scores in each row.

The dictionary method based on the World Gender Name Dictionary performs the worst, with an overall accuracy, precision, and recall around 40%. This is not surprising, considering that multiple evaluation studies have found dictionary approaches to underperform in the past (e.g., González-Bailon and Patoglou, 2015; Hartmann et al., 2019). However, based on our experience, the accuracy might be improved given a reduced approach. As will be discussed in more detail below, the World Gender Name Dictionary consists of a large sample including rare names, which lead to misclassifications when applied to texts with non-name words. The performance of the dictionary method could increase, but only if common names are included and the text is preprocessed in advance.

The application of Gender API underperforms the hand-coded survey, with its overall accuracy, precision, and recall around 50%. This is surprising considering that Gender-API operates on the basis of a relatively large database, when compared to our dictionary and machine learning approach. However, in our case only the channel names were processed by the API, while the MNB and survey method included the channel description. Future research could evaluate whether the addition of channel descriptions contributes to the Gender APIs performance, or adds distracting information that worsens the scores.

Overall, it becomes clear that the MNB machine learning approach performance is the best of the four single classifiers. Taking into account the slight variation between the test sample (*n* = 40), and the average performance across all five folds (*n* = 200), the model's accuracy, precision, and recall all score around 66%. The Brier score of about 0.05 also attests high accuracy of the predictions based on probabilities. Multinomial Naïve Bayes is known to perform well with classifying text data, especially in small samples (Kowsari et al., 2019). However, considering that many research projects will have larger samples available, which might also be more thematically focused, one can expect that the MNB approach will perform even better in these cases.

Finally, we present results of a combined weighted classifier (Kittler et al., 1998) in order to further improve the decision for one (combined) classification approach over another (Seliya et al., 2009; Liu et al., 2014). We use a combined weighted vote classifier (Dogan and Birant, 2019), aggregating the individual performance metrics of all automated classification methods into one metric, which then could serve as a further basis for choosing the best classification strategy to determining the gender of YouTube creators efficiently in large-scale data. More precisely, we assigned the final gender classification of the automated methods of each YouTube channel a vote, then weighted those votes with the overall accuracy of each method, and counted the votes in the end. The linear weighting assured that we obtain results even in those cases where each method assigns a different gender classification. When we compared the combined classifier to the multi-platform research strategy, we obtained 141 correctly classified cases. Thus, the combination of all three automated methods provided the highest overall accuracy for male and female accuracy, as well as precision for multi-agent and NA classification.

Looking at details of the performance in each gender category, we want to point out some further key insights of the methods: (1) The true value of the survey method seems to lie in its precision, where it clearly outperforms the other methods in its classification of men, women, and multi-agent channels. For men, the precision reaches 98%, meaning that 98% of male channels were correctly classified as male. (2) Interestingly, the survey and dictionary method seem to perform poorly when dealing with the No Answer-category, where they give rather moderate results. While the accuracy in this category is average, its precision of 2–4.7% is quite low. Both methods tend to give more conservative gender-estimates, which refrain from classification when no information is found, therefore increasing the number of NAs. In comparison, machine learning approaches generally tend to use any information given and will more likely estimate cases to belong to the majority groups (in our case male). (3) The Gender API approach is not designed to identify multi-agent channels. This illustrates an advantage for more adaptable dictionary approaches, which can add multi-agent identifiers (such as “we,” “us,” “our”) to already existing name-lists. (4) All methods show lower precision when predicting women vs. men. Especially with the dictionary method and MNB, only 20% of predicted female-led channels were actually led by women. Concerning the machine learning approach, this problem derives from an imbalance between the classes^8^, meaning that women are represented by a smaller number of cases in our sample and training data (Note: The accuracy is higher since it also takes correctly predicted men and multi-agent channels into account). In contrast, the survey seems very apt at classifying women both in accuracy and precision. (5) The combined weighted classifier demonstrates the probabilities of what can be achieved when multiple methods are integrated into one model. It produced a higher precision in its prediction of minority classes than the single automated methods, and increased the overall number of correctly predicted cases. Depending on the research interest, this increased performance could prove to be a vital step toward a better classification of text based social media content.

### Limits and benefits

The survey method is a cost intensive, but highly valid, and an adaptable approach to classify YouTube data. The classification questionnaire can be closely tailored to the researchers' interest, and easily allows for the inquiry into multiple variables at once (Hoßfeld et al., 2011; Biel and Gatica-Perez, 2013). Furthermore, one can implement multiple sources of information for the respondents to classify, such as pictures, text, or even audio or video material. Since human respondents can synthesize different kinds of information more easily, this permits precise categorizations. However, the researcher should be aware that this approach is time-consuming, taking into account the development and testing of the questionnaire, as well as its distribution among respondents. The median time for the classification of the channel type and potential gender of the YouTuber amounted to 28 s per case. Since this data set only consisted of 200 cases, the overall amount of time set aside for the actual classification was manageable. If, however, one was to apply the same method to a large set of data, or include further sources as stimulus for the respondents, more time would be needed. Additionally, this method relies on the availability of trustworthy respondents and meaningful names and descriptions provided by the YouTube channel. While other methods can be easily repeated in case of a mistake, this can be rather difficult for the survey method, requiring accurate survey construction and pretesting.

As mentioned, the expenditure of dictionary-based methods is highly dependent on the preexistence and availability of dictionaries, since their construction takes a lot of time and effort (González-Bailon and Patoglou, 2015; Rosenbusch et al., 2019). In our case, name-based gender identification proved a feasible strategy, since name lists are a relatively common open-source material. The World Gender Name Dictionary (Raffo, 2021) proves an extensive resource that is applicable to a wide range of countries. Therefore, its use on YouTube data can be recommended. However, as our detailed examples will show, researchers should put careful thought into the range of names, and the type of text this method is applied to. More “fuzzy” text always yields the potential to misclassify random words as names, thus adding errors into the gender score. The method is most resource effective when the likelihood of names (and only names) appearing in the text is high, as in the example of channel names. Channel descriptions can also include names, which may not be present in channel names. However, they also introduce a lot of other words, and therefore an increased probability of misclassifications. This could extend the time needed for text cleaning, such as removing stopwords in order to reduce errors. Therefore, the fit of the text to the dictionary should be assessed carefully. Furthermore, the identification of multi-agent channels made the definition of identifying words necessary. As explained before, our list of identifiers included only 9 words (e.g., “us,” “we,” “our”), which were chosen at face value. More effort could be spent to empirically identify key words that are present in YouTube channels managed by multiple people, to create a more evidence-based dictionary. However, this would rely on a database of pre-classified channels, whereas our strategy could be applied without known cases.

The implementation of Gender API is simple and time efficient (Karimi et al., 2016). Gender API applies an already trained algorithm by comparing the YouTube channel names to an unknown online web data basis, and therefore does not require text preprocessing as long as only channel names are included in the analysis (Wais, 2016). The code is made available to implement the algorithm into common programming languages (including R and python) Alternatively, the website offers a service to simply upload text columns online (e.g., using Excel or csv files) and receiving finished classification results. For evaluating the time efficiency, the API provides the duration for assessing the gender for each record in seconds. For one record the Gender API required around 20 milliseconds to assess the gender. However, in order to process large data volumes, it would be necessary to make use of a fee-requiring premium account. At the time of writing this article, the API allows 500 names to be classified per month without charge (see https://gender-api.com/).

Finally, as with all supervised machine learning approaches, our MNB model relied on the availability of a reliable, labeled dataset to train the model (Agarwal and Sureka, 2015; Parvandeh et al., 2020). In our case, the training data consisted of a dataset constructed by the authors on the basis of a multi-platform research. This approach is time intensive and requires accurate assessment of multiple sources of information. More time efficient approaches than the multi-platform research could include a classification survey as we used in our first approach, a self-reporting survey amongst YouTubers or even using commercial providers for the human-based labeling of huge amounts of data, e.g., Amazon Mechanical Turk. Once the machine learning model is established, it can be applied to new and large datasets not feasible for manual coding. This approach is especially efficient when very large samples are available, or the number of channels that have to be coded is unclear (e.g., channels are added to the dataset over time). As our example shows, the setup of a MNB classifier is relatively simple and time efficient. Since the model assumes no relation between the features, and relies on simple word count, there are few hyperparameters that have to be tuned and monitored. However, the text preprocessing is an important step before training the model and requires careful attention. In our case, the treatment of stopwords and unicodes provided challenges, as will be further explained below. Furthermore, as shown for the three automated methods, the machine learning classification can be improved through its' combination with other methods.

### Meaningfulness

The performance and cost-efficiency of methods must also be weighed against the meaningfulness and interpretability of the results, especially when sensitive subjects such as gender are involved (Wu et al., 2015; Hartmann et al., 2019). To evaluate our results, we provide an exemplarily illustration of seven YouTube channels, chosen in order to present differences and problems that occurred in our classification methods (see Table 3 for details).

**Table 3:** Exemplary results of different classification methods.

**Channel name and description**	**Ref**.	**Survey**	**Dict**.	**API**	**MNB**	**Vote**
Jana's Welt	Female	Female	NA	Male	Male	Male
**Cookie**
Hi I am Felipe. I only do YouTube and Twitch as a hobby. On this channel is actually only gaming content such as Fortnite. Have fun on my channel 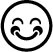	Male	NA	Female	NA	Male	Male
**Gleichberechtigt** - A self-portrait - Born in Baden in the 196x-er, studied technology at the University of Karlsruhe, graduated in 1993, employed for 4 years, then self-employed in EDP. Why not more precise?–Because state-subsidized terror executes again and again “progressive” politics of the left establishment CDU/CSU, SPD, Greens, Left and FDP and the 68'er justice finds pleasure in it–briefly because “DDR 2.0.” (…)	Male	Male	Female	NA	Multi-agent	Multi-agent
**Christelle Proudwatcher** Christelle Proudwatcher Player level 20 Server 11 | Germany 53 horses (…)	NA (lgbtqia+)	Female	Female	Female	NA	Female
**Tini and Uwe Mayer** “The Mayers on Tour”–that's Tini and Uwe Mayer–formerly from Göppingen in Baden Württemberg. Our topics: Moving into the camper and “living on the road”–travel–photography–image editing–music–lifestyle. (…)	Multi-agent	Multi-agent	Multi-agent	Male	Multi-agent	Multi-agent
**Faina Yunusova** П р и в е т ! Я х у д о ж н и к. M о я ц е л ь - п о з н а к о м и т ь в а с с с о в р е м е н н ы м и с к у с с т в о м, х у д о ж н и к а м и и и н т е р е с н ы м и и д е я м и ! П р и с о е д и н я й т е с ь !	Female	NA	Female	Female	Female	Female

Source: own calculations, texts were translated from German to English by the authors.

First of all, name-based approaches risk the misclassification of common words as given-names. The channel “Jana's Welt” [“Jana's World”] is hosted by a woman but assessed as male by Gender API. This discrepancy is based on the fact, that Gender API uses “Welt” [“World”] as sole gender indicator (excluding “Jana's” as a reference word), thus assessing a male gender with a probability of 100%. The algorithm is only referring to four examples of “Welt” in the underlying (unknown) online database, whereas usually the algorithm classifies other records based on several thousand examples. Nevertheless, as the underlying algorithm is unknown to us, the actual decision making process of Gender API remains a sort of black box. Jana's Welt was also not recognized as a female name by our dictionary approach, likely due to the possessive “s” included in the name. It remained unclassified by the dictionary method, since no names were detected. The channel does not provide a channel description that can serve as the basis for further information. Only the survey managed to classify this case correctly.

Looking at the channel “Cookie” we know that this channel is hosted by a man. We obtained no result by the Gender API (non-classified), since no name was detected. Interestingly, in this case the survey method also failed to correctly classify this channel^9^. Even though the channel description mentions the name of the YouTuber (see Table 3), the respondents reported difficulties with deciding whether “Felipe” was a male or a female name. Similar problems might arise with names that are uncommon among the German population, or that are gendered differently in different cultures (i.e., Andrea being a female name in Germany and a male name in Italy). In this case, the MNB model was the only method successful in classifying the gender correctly based on the content of the channel description.

The channel “Gleichberechtigt” is an example of a YouTuber who reveals a lot information about himself in his channel description. Coding by hand or through the survey, we could identify gender, decade of birth, education and even occupational path. However, this case also illustrates the limitations of automatic classifications. Since the channel name “Gleichberechtigt” (meaning “having equal rights” in German) does not hold any name information, the dictionary method and Gender API could not derive any classification from this information. The dictionary method however, thought it identified five female names and four male names in the channel description, and misclassified the channel as female. One concern when using large dictionaries on social media text, is that random words can be misinterpreted as names. For example, the dictionary recognizes “mehr” as a Persian name present in German records. However, “mehr” is also the German word for “more,” misinterpreted as a name in this case. Finally, the MNB misclassified the channel as a multi-agent channel, likely because the description talks about many political issues, also present in other news or party channels in our sample.

The classification methods presented in this paper aim to capture the gender self-presentation of the owners of YouTube channels. They are dependent on YouTube channels to reveal gender-relevant information within the texts or pictures representing the channel. As we have seen within our sample, many channels include the given name (or a self-chosen given name) of the YouTuber within the channel name or description, which allows for gender inference. However, these methods also have limitations when more nuanced gender-identities are concerned. One such case in our dataset is “Christelle” who identifies themselves as a demiboy^10^ in their introduction video and focuses their channel around a game called “starstable” and lqbtqia+ pride content. However, since this identity is not declared in the name or channel description, our methods mostly misclassified them as a female. Christelle is also the only apparent lgbtqia+ member in our small sample, making it difficult for machine learning approaches to consider these gender identities. Less common identities such as Christelle's will likely be underestimated in most automatic classification efforts, which should be taken into account in research design.

Tini and Uwe Mayer present an example of a multi-agent channel owned and lead by a couple. YouTube, like many other social media platforms, hosts a mix of private channels representing individuals, as well as a variety of multi-agent channels. Our sample includes channels by couples or groups of people (e.g., bands, siblings, married couples), as well as organizational channels (e.g., news outlets, TV-shows, political parties, co-operations). It can be important to distinguish between these types of channels, not only when gender is concerned, since the resources behind public or professional channels might differ significantly, therefore the number of videos, content, and views reached might also be significantly different. In terms of gender identification, these multi-agent channels provide some difficulties. In some cases, the gender of their members may be classifiable, such as with “Tini and Uwe Mayer” (see Table 3), which could be classified as a multi-agent channel by most methods, and could further be identified as consisting of a man and a woman. Using Gender API, this channel was wrongly assessed as male (with a high probability of 96%), because multi-agent channels were not included and therefore “Tini and Uwe Mayer” was read as one single male individual by the algorithm. This problem is not to be neglected, since according to the survey, out of 47 multi-agent channels in our sample, 15 were classified as multi-agent channels (groups or pairs) and 32 as non-agent channels (e.g., events, organizations).

Finally, the channel “Faina Yunusova” illustrates the problem of multi-lingual channels in our sample. Although our sample included only German YouTubers, several channels from female YouTubers used the Cyrillic alphabet. Since this YouTuber uses a given name, the dictionary method as well as the Gender API managed to classify this channel correctly as female. However, the respondents of our survey did not know the gender of the name “Faina,” neither were they able to read the Cyrillic description, and therefore did not classify this channel. Interestingly, due to the small sample size and few opportunities to compare, the MNB model interprets Cyrillic letters as being more representative of female YouTubers, and therefore classifies Faina as a woman. Furthermore, a problem arises because Cyrillic letters are represented as Unicode in our dataset (e.g., the letter и is represented as < U+0438>). The machine learning approach interprets these unicodes as words instead of letters, giving each letter more weight in the final data. Such encrypting problems resulting from multiple language use are likely common in social media data. On the one hand, authors have to decide whether these transnational identities are important for their research or not, and if more rigorous data cleaning has to be applied beforehand to remove unicodes. On the other hand, unicodes such as emojis can also yield important information for the model. For example, in our sample heart emojis were more commonly used by female YouTubers. Such gender specific use of emojis can greatly aid when using a machine learning model. Several studies concur that emoji use is especially beneficial in determining the author's gender (Wolf, 2000; Chen et al., 2018; Beltran et al., 2021).

### Ethical challenges

Based on our study, we want to contribute to existing research by highlighting some ethical challenges discussed in social sciences, which may arise from the inference of gender from YouTube data. One major pitfall of applying automatic classification methods involves the (re-)production of gender stereotypes (Dinan et al., 2020). The MNB machine learning approach is especially at risk of such behavior, since all words of the channel description are processed and assigned with a certain gendered probability, based on the information the model derives from the training data. However, if the training data finds men to be mainly dealing with politics, and women with beauty issues, the attributed words will then be associated with stereotypic gender categories. This reproduction of statistical differences is known as statistical discrimination (Arrow, 1974) in the social sciences, and is related to profound consequences, especially when looking at members of small or vulnerable groups (Leavy, 2018). At this point, it seems plausible that representatives of the lgbtqia+ community, for example, would have to face higher risks of stereotypical gender classification or even misclassification, since randomly selected training data presumably does not rely on valid information in this realm. With respect to our own study, we find an unwanted association between Cyrillic letters and women, as well as a higher association of men with video games (see Meaningfulness for examples). While the first observation is bound to the process of stereotypical classification and calls for more rigorous pre-processing of the text data, the second observation might represent both aspects at the same time: the result of biased training data and/or an interesting finding. This underlines the need for a thoughtful interpretation of results, a diligent evaluation of the field of application, sample selection criteria and the fit of research question to the selected design^11^.

## Discussion

The purpose of the present paper was to compare four text-based classification methods in order to assess the gender of German social media content creators. By using the example of a random sample of 200 YouTube channels, we compare a classification survey, a name dictionary method with the World Gender Name Dictionary as a reference list, an algorithmic approach using APIs of the website gender-api.com, and a Multinomial Naïve Bayes (MNB) machine learning technique. With the help of these different approaches, we identified gender attributes based on YouTube channel names or descriptions, and contrasted our results with a reference dataset to evaluate them. The reference dataset contained all information available on each channel using a multi-platform research strategy (Jordan, 2018; Van Bruwaene et al., 2020), including YouTube channels, Facebook and Instagram profiles, Twitter accounts, Google and Wikipedia data.

Our main conclusions concerning the pros and cons of each method are summarized in Table 4. They reveal that the MNB machine learning technique performs the best within our sample of single classifiers, since the model's accuracy, precision and recall all score highly (~66%). However, the presence of a training sample is required, and one should be aware of stereotypical classification problems (see Ethical challenges). Second best is the online survey method, with accuracy, precision, and recall scores around 60%, especially when multiple information sources are combined. Here, one should take into account that this method is rather time consuming and possibly in need of a large number of respondents. Using gender-api.com underperforms the classification survey, with its overall accuracy, precision, and recall around 50%. Nevertheless, this method is simple, time efficient, and the use of resources is quite low when small data volumes are processed. The dictionary method based on the World Gender Name Dictionary performs the worst, with its overall accuracy, precision, and recall around 40%. Here the performance is especially low when the text includes a lot of non-name noise. Finally, with respect to the combined voted classifier (Kittler et al., 1998), we observe that the integration of all three automated classification techniques would yield even better results on gender classification outcomes than single classifiers (Khaled and Ali, 2020). These improved results are achieved because the weaknesses of each single classification method is compensated for in the combined metric, and should therefore be noted in future research.

**Table 4:** Overview of the results.

	**Class survey**	**Name dictionary**	**Gender API**	**MNB**	**Weighted vote**
Performance	High, especially with multiple sources combined	Low, especially when text includes a lot of non-name noise	Moderate, depending on the noise within the text	High, especially for large samples and majority groups	High, even for minority groups
Limits and benefits	Time consuming, though with little requirements	Very low when already present dictionary (e.g., WGND) are used; text preprocessing might be necessary	Very low when small data volumes are processed, large volumes require a fee	Presence of a training sample is required. Otherwise, low number of parameters	Low, but dependent on existing models that are included and their requirements
Meaningfulness	High, though dependent on the openness of answers available to respondents	Dependent on the noise within the text, and number of words misidentified as names; identifies names can be accessed	Dependent on the noise within the text, and number of words misidentified as names; high accessibility of feature probabilities	High accessibility of feature probabilities, chance of stereotypical classification	Dependent on previous models included in the vote and their meaningfulness
Ethical challenges	Reinforcement of stereotypes based on individual experiences of respondents	Reinforcement of stereotypes based on country-specific name lists	Reinforcement of stereotypes based on structure of unknown online reference data	Reinforcement of stereotypes based on bias in the training data	Reinforcement on stereotypes and misclassification of included models

Source: own illustration.

We have shown that the inference of gender categories from YouTube channel names and descriptions is very well-possible, given some limitations. At best, about two thirds of channels will be correctly classified, depending on the methods used. In our case, the combination of automated classification techniques outperformed the other methods. The availability of a valid training data set is key to the quality of the outcome, and decisive for the level of detail achieved in this kind of research. Nevertheless, our study also shows that the final classifications do have their biases. They overestimate the presence of men on YouTube, for example due to false name-classification. Minority groups such as women, and more extensively non-binary gender identities, remain underrepresented or undetectable by the methods presented.

In light of our results, we want to offer some further thoughts on the use of (automated) classification methods for the social sciences. Overall, the classification of socio demographic characteristics is a key agenda for this field of study, because it allows scholars to explore the social contexts of online behavior. If we remain blind to the enhanced functionalities of gender, but also age, ethnicity, or education in online spaces, we risk overlooking the social structures and inequalities in contemporary digitized societies (Wagner et al., 2015; Karimi et al., 2016). Because the lack of information on vulnerable groups (e.g., women or non-binary individuals) and the hurdle to gather other crucial socio demographic characteristics (e.g., education or migrant background) opens a window of stereotypic digital narratives, preventing to tackle traditional patriarchal images associated with prestige, reason and power (Sobande, 2017; Fosch-Villaronga et al., 2021). This becomes even more relevant when we interlink social science theories and empirical findings to the emerging research field of machine learning. Against this background, we want to encourage scholars to further elaborate on text-based classification methods of social media data in future research:

- To date, we see great potential in automated classification methods in social science matters, since the results achieved by these relatively simple approaches are impressive and especially eligible for processing great volumes of data. However, this paper focused on gender classification which is more easily detectable and assignable compared to ethnicity, educational background, or occupational affiliation for example. Therefore, we also see some credible challenges, which should be subject to future studies.
- In light of key empirical findings and existing challenges, we would strongly recommend the combination of the application of ML based text classification with other methods, such as self-reporting surveys or classification surveys in order to generate precise data that allows the investigation of (re-)producing social inequalities in platform-based societies (van Dijk, 2020).
- We encourage researchers to actively counter steer the invisibility or misrepresentation of information within automated classifications of social media data, especially when marginalized groups are involved. At this point, more research is needed to find ways to reduce the bias present in all methods discussed above. This again indicates a need for elaborating on existing classification methods, and might even point toward the requirement to integrating other methods, for example in-depth qualitative interviews, in order to tackle blind spots and achieve a solid interlinkage of theory production and empirical research.
- Based on our findings, the presence of Emojis, multiple languages (which might provide encoding issues), multi-agent channels, and “noisy” text in the YouTube channel descriptions present hurdles to automated classifications. We have outlined some strategies to mitigate these problems in the presented study. However, these topics also warrant more methodological inquiry.
- Finally, we are convinced that further research should be dedicated to the valuation of multiple information sources available on YouTube and other social media platforms such as Instagram or Tiktok. In the present study, we use the channel names and descriptions as the only data source. Nevertheless, video content, channel profile pictures, audio and video data are further valuable sources of information, which might still be in their early days of development, but already yield some promising and trendsetting approaches.

## Data availability statement

The data used in this paper is available on GitLab (project id 37844399).

## Author contributions

LS and RV revised the project, the main conceptual ideas, and proof outline. LS prepared the reference data set, performed the dictionary categorizations, and designed the machine learning model. CB designed the SoSci survey and analyzed the survey data. N-SF performed the API categorizations. LS, RV, CB, and N-SF contributed to the initial submission of the manuscript. RV, CB, and N-SF produced the final version of this text, which was approved by all authors.

## Funding

The work on this study was supported through a research grant of the German Research Foundation (DFG, Grant Number VE 375/10-1).

## Conflict of interest

The authors declare that the research was conducted in the absence of any commercial or financial relationships that could be construed as a potential conflict of interest.

## Publisher's note

All claims expressed in this article are solely those of the authors and do not necessarily represent those of their affiliated organizations, or those of the publisher, the editors and the reviewers. Any product that may be evaluated in this article, or claim that may be made by its manufacturer, is not guaranteed or endorsed by the publisher.

## Footnotes

^1^We refer to gender rather than to the biological sex, including expressions of gender roles and norms, as well as gender-specific representations in text-based descriptions, which we found on the web, viewing women, men and non-binary individuals as social categories and culturally constructed subjective identities (Oakley, 2016; Leavy, 2018). Here, we understand that social categories refer to the common identification with a social collectivity that creates a common culture among participants concerned, thus effecting individuals' self-perception and (online) behavior.

^2^The sociodemographic composition of YouTube creators is rarely studied. However, our sample seems to be in line with existing studies. For example, Debove et al. (2021, p. 4 ff.) found the percentage of women, men, and institutions among their sample of French science channels to be 12, 64, and 21%. Wegener et al. (2020) have roughly 17% female, 53% male, and 30% institutional creators in their study of top-rated German YouTube channels and Regueira et al. (2020) observed 10% women, 60% man, and 30% institutional creators in top-listed Spanish YouTube channels.

^3^This relatively high viewership and distribution inequality are related to the fact that our random sample includes a very popular YouTube channels of a German TV Show (“Berlin Tag und Nacht”). However, most other channels in our sample present few views. Nevertheless, other studies on YouTube have shown that an unequal distribution of viewership is typical for this platform (Tang et al., 2012; Zhou et al., 2016).

^4^The NB is a probability-based approach that calculates the probability of a certain document to be part of a specific class given its features. In our case, we calculate the probability of a channel description to belong to one of four gender categories given the words and emojis is based on the following equation: $P\left(c_{k}|x\right)=P\left(c_{k}\right)\;\times \;\frac{P\left(x|c_{k}\right)}{P\left(x\right)}$. *P*(*c*_*k*_|*x*) being the conditional probability of the occurrence of a category given the existence of a vector of features *x*. *P*(*c*_*k*_) is the general probability of the occurrence of the category, *P*(*x*|*c*_*k*_) being the conditional probability of a certain word belonging to a category and *P*(*x*) being the probability of the occurrence of the feature x. Naïve Bayes assumes that all features are independent from one another (Lewis, 1998).

^5^Our machine learning model was not able to deal with non-binary cases due to the limited number of cases. We therefore decided to classify this single object as NA (not available), whilst formatting the data set. As final outcome categories for the machine learning model, we keep “female,” “male,” “multi-agent” and “NA”.

^6^In our study, the sample size of the dataset was relatively small, which is not ideal for machine learning approaches, but also not uncommon for social scientists working with data donations through surveys (Molyneaux et al., 2008; Muñoz Morcillo et al., 2019; Chen et al., 2021; Debove et al., 2021).

^7^Machine learning models would be capable of integrating alternative procedures such as image classification, speech recognition, or face recognition for obtaining gender assignment estimates (Hinton, 2012; Balaban, 2015). However, problems with these methods remain far from being solved. For example, 2-D image representations of human faces exhibit large variations due to illumination, facial expression, pose, the complexity of the image background, and aging variations (Kasar et al., 2016). Moreover, image examples available for training face recognition machines are limited which makes the task of characterizing subjects difficult.

^8^Imbalanced datasets present a challenge to machine learning algorithms for which various strategies exist (Weiss, 2013). A common way is to resample the training dataset by either undersampling the majority class or oversampling the minority class (Chawla et al., 2002; Agrawal et al., 2015). In our case we refrained from this step since (1) our imbalance is only moderate with the highest proportional difference being between the male and the NA class. (2) This class imbalance in our data seems to mimic real life as it is very similar to previous findings in other studies (see Regueira et al., 2020; Wegener et al., 2020; Debove et al., 2021) and thus gives the machine learning algorithm further information about the natural occurrence of each class. (3) We used a modest data set of *N* = 200 (see chapter 3). Undersampling the male class would risk the loss of valuable information which is needed for a valid classification and oversampling the three minority classes risked problems of overfitting the model, since the relative number of cases was rather low.

^9^However, in other examples of fictious names the survey approach might be more powerful in classifying gender information. One example is the use of names, associated with a gender, such as “Legolas” or “Yoda” as prominent (science) fiction characters from Lord of the Rings and Star Wars.

^10^The term demiboy describes a non-binary gender identity with predominantly male characteristics.

^11^In terms of sample selection criteria and field of application, our study points towards to some additional ethical challenges, which are not central to the present paper, yet interesting to discuss in future research. Our sample includes several YouTube channels which feature minors under the age of 10, and although all information is made publicly available (most likely by their parents or agents), these children are vulnerable subjects of research as their consent to the publication of the material cannot be taken for granted. This calls for a broader discussion on how to handle the passive participation of individuals portrayed in YouTube channels in social science research, although scholars do not technically require the direct consent of the subjects, nor is it (yet) necessary to inform them about the study.
